# AMPK and Polycystic Kidney Disease Drug Development: An Interesting Off-Target Target

**DOI:** 10.3389/fmed.2022.753418

**Published:** 2022-01-31

**Authors:** Michael J. Caplan

**Affiliations:** Department of Cellular and Molecular Physiology, Yale University School of Medicine, New Haven, CT, United States

**Keywords:** Autosomal Dominant Polycystic Kidney Disease, adenosine monophosphate-stimulated protein kinase, metabolism, mTOR, CFTR, metformin

## Abstract

Autosomal Dominant Polycystic Kidney Disease is a genetic disease that causes dramatic perturbations of both renal tissue architecture and of a multitude of cellular signaling pathways. The relationship between the products of the genes whose mutations cause polycystic kidney disease and these signaling pathways remains difficult to determine. It is clear, however, that cellular metabolism is dramatically altered in cells that are affected by polycystic kidney disease mutations. Adenosine monophosphate-stimulated protein kinase is a master regulator of cellular energy use and generation pathways whose activity appears to be perturbed in cells affected by polycystic kidney disease. Furthermore, modulation of this enzyme's activity may constitute a promising approach for the development of new therapeutics for polycystic kidney disease.

## Introduction

The clinical presentation of Autosomal Dominant Polycystic Kidney Disease (ADPKD) is most notable for the massive enlargement of the kidneys that affected individuals often experience over the space of decades (1). This dramatic transformation is produced through the development of numerous fluid-filled cysts that derive from nephron epithelial cells and whose expansion compresses and damages the surrounding normal parenchyma, resulting in end stage renal disease in ~50% of ADPKD patients. ADPKD is the most common life-threatening genetic disorder, affecting ~1:1,000 individuals independent of race, ethnicity, and gender. Mutations in either of two genes, *PKD1* and *PKD2*, account for ~95% of cases of ADPKD (2). Consistent with the condition's autosomal dominant mode of inheritance, patients generally carry one wild type and one mutant allele of *PKD1* or *PKD2*. While the mechanisms that initiate cyst formation remain the subject of investigation and debate, there is strong evidence that the stochastic acquisition of “second hit” mutations in the wild type *PKD1* or *PKD2* allele can play an important role in catalyzing the processes that lead to cyst development (3–5). According to this model, an individual epithelial cell in which a second hit mutation occurs becomes mitotically active and the proliferation that follows populates the wall of the resultant cyst. These cells also acquire a secretory phenotype, which contributes to the cyst's expanding fluid volume (6).

While the disruption of kidney architecture that ADPKD produces is anything but subtle, the mechanisms that connect mutation of the *PKD1* and *PKD2* genes to this profound structural disturbance remain obscure. The precise physiological functions of polycystin-1 (PC1) and polycystin-2 (PC2), the proteins encoded by *PKD1* and *PKD2* respectively, have yet to be elucidated, more than two decades after these genes were identified. Similarly, the processes through which the absence of functional PC1 or PC2 leads to the formation of cysts have not been completely unraveled. The mystery that shrouds the biological roles of PC1 and PC2 is not attributable to a lack of evidence for candidate signaling pathways in which they may participate. On the contrary, efforts to understand the cellular and molecular basis of ADPKD pathogenesis have been complicated in part by the overabundance of cellular processes that appear to be connected to, regulated by or responsible for governing some aspect of polycystin protein activity. It is clear that perturbing the expression of PC1 and PC2 extends ripples of downstream effects through a multiplicity of effectors (7, 8). It is not clear, however, which of these effectors are most proximate to the polycystin proteins themselves nor is it understood whether or how alterations in the messages communicated by one or more of these effectors can initiate cyst formation.

The absence of a causative link between the polycystin proteins and a single, direct and definitive explanation for cystic disease has been an impediment to the development of targeted therapeutics. Efforts to discover highly specific and efficacious drugs typically begin with assays that have evolved from a deep understanding of the relationship between a target and a disease. In the case of ADPKD such efforts have, of necessity, focused on intervening in processes that contribute to cyst formation, proliferation or expansion, even if the connections between those processes and the polycystin proteins are not clearly established. Thus, while the V2R vasopressin receptor is not thought to be an immediate upstream or downstream consort of the polycystin proteins, its influence on the elevated cAMP levels that clearly contribute to cystogenesis is exploited by Tolvaptan, the first drug that has been approved for the treatment of ADPKD (9, 10). Similarly, while the mechanisms that account for elevated mTOR activity in ADPKD are not fully understood, very convincing pre-clinical data from animal studies demonstrating that mTOR inhibition ameliorates cystic disease served as the basis for human trials of this approach (11–21). Until such time as a consensus understanding of the physiological roles of the polycystin proteins emerges, it is likely that progress in the development of new ADPKD therapeutics will resemble these efforts to target molecules and pathways that contribute to the cystic phenotype even if the biological basis of their connections to the functions of the polycystin proteins is obscure.

## AMPK: A New Target for ADPKD Drug Development

**Figure 1:**
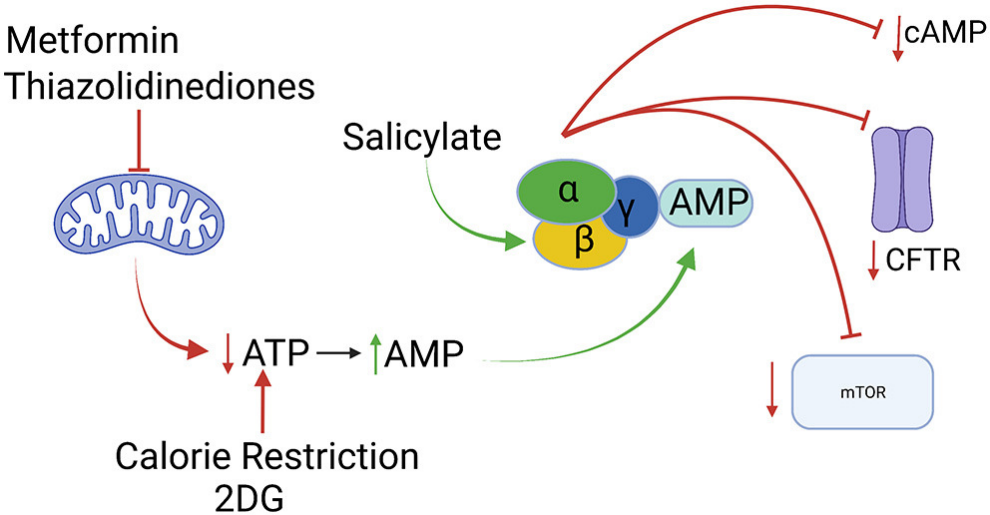
Activation of the AMPK enzyme, depicted as a heterotrimer of α, β and γ subunits, can result in inhibition of of the mTOR pathway, of CFTR-mediated fluid secretion and of cAMP accumulation. AMPK activity can be stimulated indirectly by interventions that reduce ATP production [metformin, thiazolidinediones, 2-deoxy-D-glucose (2DG), calorie restriction] or directly (salicylate). All of interventions slow disease progression in animal models of ADPKD. Created with Biorender.com.

When considered through the lens of this rather broadminded approach to target identification, the cellular energy sensor and regulator adenosine monophosphate-stimulated protein kinase (AMPK) comes into focus as an extremely interesting candidate for ADPKD drug development (8). As its name implies, the kinase activity of AMPK is stimulated when AMP levels rise (22). This occurs when ATP levels fall, thanks to the function of the ATP:AMP transphosphorylase, which converts two molecules of ADP into one ATP and one AMP. As befits a master regulator of cellular energy utilization and generation, activation of AMPK leads to inhibition of energy-intensive processes and upregulation of energy generating processes. Among the energy-intensive pursuits that are shut down by AMPK are several that are relevant to processes that drive cyst expansion in ADPKD. Fluid secretion into the ADPKD cyst lumen depends upon active electrolyte transport that requires, at least in part, the participation of the cystic fibrosis transmembrane conductance regulator (CFTR) chloride channel (23, 24). AMPK-mediated phosphorylation of CFTR inhibits its channel activity, thus reducing energy-consuming transepithelial ion fluxes (25, 26). By phosphorylating the tuberin protein (TSC2), AMPK stimulates the GTPase-activating protein (GAP) activity of the TSC1/2 tuberous sclerosis complex (27). This in turn leads to conversion of the small GTP binding protein Rheb from its GTP bound form to its GDP bound form, which results in the inhibition of the mTORC1 complex and its kinase activity. Inhibition of mTOR suppresses protein synthesis and cell proliferation (28), both of which are energy-demanding processes that contribute to ADPKD cyst formation. Interestingly the chromosomal location of the *TSC2* gene is extremely close to the 3' end of the *PKD1* gene. Mutations that compromise the expression of both the TSC2 and PC1 proteins produce ADPKD with a severe, early onset course, reinforcing the importance of TSC2 and the mTOR pathway that it regulates in the development of the cystic phenotype (29). Finally, in at least some cell types activation of AMPK may reduce cAMP levels, since AMPK phosphorylates and activates the cAMP degrading enzyme phosphodiesterase 4B (30) (Figure 1). It is also worth noting that cilia, which play an important role in pathways associated with polycystin-related signaling, also exert influence on the regulation of AMPK activity (31, 32). Because of its potential to decrease the contributions of three key pathways to cyst expansion, Takiar et al. proposed in 2011 that AMPK could constitute a novel and accessible target for ADPKD therapeutic development (33).

AMPK is a heterotrimer, composed of an α-subunit that possesses the kinase activity, a γ-subunit that binds to AMP and a β-subunit that serves as the structural connection between α and γ. There are two isoforms each of the α and the β-subunits, and three isoforms of the γ-subunit (34). Renal epithelial cells predominantly express the α1 and β1-subunit isoform proteins, as well as both the γ1 and γ2 isoforms of the γ-subunit protein (35). Interaction with AMP induces a conformational change in the γ-subunit that is communicated to the α-subunit, as a result of which the α-subunit becomes a substrate for triggering phosphorylation by LKB1 or CAMKK, both of which can serve as upstream activators of the AMPK kinase (36). This phosphorylation, which occurs at Thr172, can be detected by western blotting using a phospho-specific antibody directed against phospho-AMPK (pAMPK) and the signal obtained in such assays is widely used as a surrogate for the level of AMPK kinase activity.

Perturbations in cellular energetics are features of a wide variety of diseases, including diabetes and cancer. Because of its central role as a regulator of the cell's energy economy, AMPK has been viewed as a potentially useful tool that could possibly be brought to bear to correct these perturbations and thus to treat the diseases that cause them (37). In order for the useful properties of AMPK to be exploited for therapeutic purposes, it is necessary to identify safe and well-tolerated treatments that effectively induce AMPK kinase activation. Fortunately, this can be achieved through several different direct and indirect approaches.

## ADPKD and Cellular Energetics in ADPKD

Although AMPK was first proposed and explored as a target for ADPKD therapeutic development in 2011 (33), the first demonstration that AMPK activity might be inappropriately suppressed in the context of ADPKD was provided in a landmark paper by Rowe et al. (38). These investigators discovered that cells that lack expression of PC1 exhibit a profound alteration in their metabolic processes. Even in the presence of oxygen, the highly proliferative cells that lack PC1 prefer to generate ATP via glycolysis. This behavior resembles the Warburg effect, which is characteristic of many types of highly proliferative tumor cells (39). In the case of the cells that lack PC1 expression, this metabolic dysregulation leads not only to a suppression of oxidative phosphorylation in favor of glycolysis, but also to a surprisingly large increase in the rate of glycolysis and ATP production. The levels of ATP in cells that lack PC1 are sufficiently elevated to result in the suppression of the activity of AMPK, which is normally triggered when ATP levels fall. This behavior was observed both in cultured cells and in renal tissue for an ADPKD mouse model (38). In an effort to exploit the metabolic vulnerability created by this switch to glycolysis, these investigators treated mice with 2-deoxy-D-glucose (2DG), which is a competitive inhibitor of an early step in glycolysis. Treatment with 2DG slowed the progression of cystic disease and, consistent with its capacity to interrupt ATP production in the glycolysis-dependent ADPKD cells, it also led to the activation of AMPK (38, 40). Thus, it is tempting to hypothesize that the beneficial effects produced by 2DG in the context of cystic disease are referenceable at least in part to its stimulatory effect on AMPK.

## Indirect AMPK Activators

Calorie restriction is another indirect but simple and very well-tolerated intervention that leads to AMPK activation. In the context of ADPKD, Warner et al. showed that food restriction dramatically slowed cyst development in a mouse model of ADPKD (41). This effect was “dose dependent,” in that the degree of cystic disease suppression was well-correlated with the extent of the food restriction, which ranged from 10 to 40% in this study. Perhaps most striking was the observation that the imposition of calorie restriction was able to at least partially reverse established cystic disease. While many mechanisms could contribute to the suppression of cystic disease by food restriction, it is interesting to note that food restriction might be expected to produce AMPK activation since it should reduce the availability of the substrate molecules that are required to fuel cellular energy production. In fact, in renal tissue from food restricted animals AMPK activity is substantially increased, as evidenced by increased levels of pAMPK. In addition, the activity of the mTOR pathway is reduced in these animals, consistent with increased AMPK activation. Thus, it is possible that some or all of the influence of food restriction on the course of cystic disease progression is attributable to its capacity to activate AMPK.

Calorie restriction and 2DG deny cells the high energy substrates that they require to produce ATP, thus lowering ATP levels, raising AMP levels and producing AMPK activation. A number of insulin-sensitizing drugs that are widely used to treat Type 2 diabetes, the metabolic syndrome and polycystic ovary syndrome, also reduce ATP levels, elevate AMP levels and produce consequent AMPK activation. These drugs do not act by reducing the availability of high energy substrates but instead by interfering with the capacity of mitochondria to carry out the oxidative phosphorylation that liberates the energy embedded in those substrates. Metformin has been used for this purpose for more than 60 years. The precise mechanism through which metformin produces reduced ATP levels and increased AMP levels is not entirely clear, but it is generally thought that metformin inhibits the mitochondrial electron transport chain by blocking the function of Complex I (42). It is also important to note that metformin appears to affect numerous cellular processes and that therapeutic doses of metformin administered to treat Type 2 diabetes may not be sufficient to incite AMPK activation *in vivo* (30, 37, 43–46). It is likely, therefore, that its insulin-sensitizing and hypoglycemic effects are not directly related to its capacity to activate AMPK. Thiazolidinediones are another class of insulin-sensitizing drugs that produce multiple effects, among which are both inhibition of mitochondrial Complex I and AMPK activation (47, 48). It has recently been shown that, at least in the liver, the elevated AMP produced by metformin treatment can act to inhibit adenylate cyclase and thus prevent hormone-stimulated increases in cAMP (44). It is possible, therefore, that in addition to possessing the potential to activate AMPK, metformin may also have the ability to suppress cAMP levels. Thus, at least in theory, metformin may be able to influence three pathways that are thought to be critical to cyst progression. Two of these pathways (mTOR and CFTR) could be suppressed by indirect metformin-induced activation of AMPK, while the third (cAMP) could be the product of indirect metformin-induced elevation of AMP and consequent inhibition of adenylate cyclase or of direct stimulatory effects of AMPK on the activity of phosphodiesterase 4B.

Because of its well-established safety profile and its possible capacity to inhibit cystogenesis through AMPK activation, Takiar et al. tested the effects of metformin treatment in cell culture systems that recapitulate aspects of ADPKD-related processes as well as in two rapidly progressing orthologous ADPKD mouse models (33). In all of these experimental settings, metformin treatment inhibited pathways associated with cyst development and slowed the progression of cystic disease. These results have since been recapitulated in other animal models of renal cystic disease, including zebrafish (49), mice (50) and pigs (51). Metformin also slowed hepatic cyst development in a rodent model of polycystic liver disease (52). It is important to note that one study employing an orthologous mouse model of ADPKD did not detect a suppressive effect of metformin treatment on cyst development (53). It will be important to understand the basis for these discrepant results, since illuminating differences in mouse strain susceptibilities, dose administration, pharmacokinetics, etc. might reveal important factors that should be considered if metformin were to be developed as a therapeutic for patients with ADPKD. At the other extreme, the data obtained with metformin and 2DG, alone or in combination, in the pig system was especially striking, since each of these treatments impressively suppressed the dramatic cystic pathology that is characteristic of this highly orthologous model of ADPKD (51). Preclinical studies have tested two thiazolidinediones, pioglitazone and rosiglitazone, in non-orthologous rodent models of PKD and found that both compounds reduced the progression of cystic disease (54–56).

The well-understood safety profiles of metformin, pioglitazone and rosiglitazone, coupled with the accumulated promising pre-clinical data, has inspired the initiation of several small clinical trials designed to assess the safety of these compounds in ADPKD patients (57–59). Two of these have recently concluded and demonstrated that both metformin and pioglitazone are safe and well-tolerated in ADPKD patients (57, 60, 61). The TAME metformin trial employed the relatively high doses of the drug that are administered to treat polycystic ovary syndrome (60). Neither study was adequately powered to provide an assessment of efficacy. Thus, a determination as to whether metformin or thiazolidinediones can produce clinical benefit for ADPKD patients will await the results of future larger trials. One such large trial, administered through the University of Queensland in Australia, is currently enrolling 1,164 patients with rapidly progressing ADPKD. This Phase 3 study, “Implementation of Metformin theraPy to Ease Decline of Kidney Function in Polycystic Kidney Disease” (IMPEDE-PKD), will test the efficacy of 2 years of treatment with a slow-release form of metformin (61). The results of this study, which is expected to conclude in 2026, should provide a valuable assessment of metformin's promise as an ADPKD therapeutic.

## Direct AMPK Activators

The interventions that have been discussed up to this point achieve AMPK activation indirectly by interfering with cellular energy production. There are also a number of AMPK-interacting compounds whose administration produces direct effects on this enzyme's kinase activity. Salicylate, the active ingredient in aspirin, physically binds to the AMPK β-subunit and induces phosphorylation-independent AMPK activation (37, 62, 63). A recent very promising study showed that administration of salsalate, which is metabolized into salicylate, produced impressive suppression of cystic disease in an orthologous mouse model of ADPKD (53). In light of its very long history as a component of the pharmacopeia and its excellent safety profile, these observations should hopefully be translatable into a human clinical trial in the near future.

Another direct, but potentially more problematic, intervention involves the administration of chemical compounds that can mimic the capacity of AMP to bind to the AMPK γ-subunit and initiate the consequent conformational changes that are prerequisites for activating phosphorylation of the α-subunit by LKB1 or CAMKK. One such compound, AICAR, has been used extensively in research studies that have helped to elucidate the physiological consequences of AMPK activation *in vitro* and *in vivo* (64). MK-8722, a compound that employs this same mechanism to achieve AMPK activation, was tested as a potential treatment for diabetes and diabetic sequelae (65, 66). Administration of this compound to rodent and monkey models produced impressive improvements in glucose tolerance and also ameliorated aspects of diabetic nephropathy. Importantly, however, exposure to this compound produced cardiac hypertrophy. As a result, concerns have been raised as to the safety of chronic administration of direct AMPK activators that share this mechanism of action.

## Discussion

It is not clear whether the activity of AMPK is directly regulated by some aspect of polycystin protein function. It is clear, however, that AMPK modulates the activity of several pathways that participate in ADPKD pathogenesis. Furthermore, a number of metabolism-related therapeutic approaches that have shown promise in preclinical models of ADPKD induce AMPK activation and may achieve their beneficial effects in slowing cyst development through their influence on AMPK (8). A great deal of work remains to be done to determine whether and how AMPK responds to polycystin-related signals. It is clear, however, that AMPK is an interesting and attractive potential target for the development of safe and effective ADPKD therapeutics.

## Author Contributions

MC wrote the manuscript and is entirely responsible for its content.

## Funding

Our research work on metabolism in ADPKD was supported by NIH RC2 grant DK120534.

## Conflict of Interest

The author declares that the research was conducted in the absence of any commercial or financial relationships that could be construed as a potential conflict of interest.

## Publisher's Note

All claims expressed in this article are solely those of the authors and do not necessarily represent those of their affiliated organizations, or those of the publisher, the editors and the reviewers. Any product that may be evaluated in this article, or claim that may be made by its manufacturer, is not guaranteed or endorsed by the publisher.
